# Decreased expression of JAK1 associated with immune infiltration and poor prognosis in lung adenocarcinoma

**DOI:** 10.18632/aging.202205

**Published:** 2020-12-15

**Authors:** Jinlian Cai, Hong Deng, Linlin Luo, Luxia You, Huitian Liao, Yan Zheng

**Affiliations:** 1 Jiangxi Key Laboratory of Molecular Medicine, The Second Affiliated Hospital of Nanchang University, Nanchang 330006, China; 2 Second Department of Respiratory Disease, Jiangxi Provincial People's Hospital Affiliated to Nanchang University, Nanchang 330006, China; 3 Financial Department, The Second Affiliated Hospital of Nanchang University, Nanchang 330006, China; 4 Department of Respiratory Disease, The First Affiliated Hospital of Nanchang University, Nanchang 330006, China; 5 Pharmacy Department, Jiujiang Hospital of Traditional Chinese Medicine, Jiujiang 332000, China

**Keywords:** JAK1, lung adenocarcinoma, prognosis, immune infiltration, M1 macrophages

## Abstract

Janus kinase 1 (JAK1) is a member of the JAK family, which plays an essential and non-redundant role in tumorigenesis. However, the potential role of JAK1 in immune infiltration and prognosis of lung adenocarcinoma (LUAD) remains unclear. The mRNA expression and methylation level of JAK1 in LUAD were examined using the Oncomine and The Cancer Genome Atlas (TCGA) databases, respectively. The correlations between JAK1 expression and its methylation level and clinicopathological parameters were analyzed. The Kaplan–Meier plotter database was used to evaluate the prognostic value of JAK1 in LUAD. The signaling pathways associated with JAK1 expression were identified by performing a GSEA. The CIBERSORT and TIMER databases were used to analyze the correlations between JAK1 and tumor-infiltrating immune cells. In addition, the JAK1 expression and proportion of immune cells in LUAD cell lines were analyzed. The JAK1 expression was remarkably decreased in patients with LUAD and significantly correlated with the clinical features of patients with LUAD. The JAK1 methylation level was increased and negatively correlated with its mRNA expression. A decrease in JAK1 expression was correlated with poor prognosis. The results of GSEA showed that cell adhesion, tumorigenesis, and immune-related signaling pathways were mainly enriched. JAK1 was positively associated with tumor-infiltrating immune cells, and the results of CIBERSORT analysis suggested that JAK1 was correlated with monotypes and M1 macrophages. The results of the TIMER database analysis confirmed that JAK1 was closely associated with the gene markers of M1 macrophages. Thus, JAK1 may serve as a potential prognostic biomarker in LUAD and is associated with immune infiltration.

## INTRODUCTION

Lung cancer is one of the most common types of malignancies worldwide and is the leading cause of cancer-related mortality. According to Globacan, more than 1 million patients die from lung cancer worldwide annually [1, 2]. Lung adenocarcinoma (LUAD) is a histological type of non-small cell lung cancer that accounts for a large proportion of cases with this disease [3]. Among the major malignancies, the economic burden of LUAD remained high for decades [4]. With the accumulation of studies on the etiology and pathogenesis of LUAD, the immune response is considered crucial to the development and progression of LUAD; this means that an improvement in the tumor immune response could be beneficial for LUAD patients, thus improving the clinical symptoms and overall survival (OS) [5]. Immunotherapy has been recently developed and widely used in patients with lung cancer, including cytotoxic T lymphocyte-associated antigen 4 inhibitors and immune checkpoint inhibitors [6–8]. Despite the advancements in targeted therapy and immunotherapy, the prognosis of patients with LUAD remains poor [9]. In the era of precision medicine, it is necessary to elucidate the relationship between LUAD and immune infiltration and explore a reliable immune-related biomarker that can predict the prognosis of LUAD and become a novel target for LUAD immunotherapy.

The Janus kinase (JAK) family is one of the families of non-receptor tyrosine kinases [10]. These JAKs are critically involved in cell growth, hematopoiesis, and immunoregulation by participating in the signal transduction of hematopoietic cytokines and growth factors [11]. JAK1 is a member of the JAK family, which plays an essential and non-redundant role in the proliferation, differentiation, and metastasis of cancer cells by mediating the expression of interleukin-6 (IL-6)/JAK1/signal transducer and activator of transcription-3 (STAT3) or JAK1/phosphatidylinositol-3-kinase (PI3K) signaling pathway [12, 13]. JAK1-deficient or mutant cell lines show greater tumorigenicity, which has been reported in patients with hepatocellular carcinoma, acute lymphoblastic leukemia, lung cancer, and gastric cancer [14–16]. A previous study suggested that JAK1 mediates many immune regulatory processes, including those that are involved in tumor-driven immune escape, cancer immunosuppression, and sustained inflammation in the tumor microenvironment [17]. Programmed death-ligand 1 induced by tumor-associated macrophages promotes the progression of lung cancer through the JAK1/STAT3 signaling pathway, and the JAK1 inhibitor significantly improves the efficacy of anti-programmed cell death protein 1 immunotherapy in pancreatic cancer by inhibiting the activation of cytotoxic T lymphocytes [18, 19]. Moreover, a lower JAK1 expression is associated with T-cell infiltration and a poor prognosis in breast cancer [20]. However, the potential role of JAK1 in tumor immune infiltration and the prognosis of LUAD remains unclear.

Since early December 2019, coronavirus disease 2019 (COVID-19), which first occurred in Wuhan City, Hubei Province, China, is among the types of pneumonia with unknown etiology [21]. It is caused by a novel positive-sense RNA virus, named severe acute respiratory syndrome coronavirus 2 (SARS-CoV-2), which belongs to the *Betacoronavirus* genus in the *Coronaviridae* family [22]. The outbreak of COVID-19 was declared as a public health emergency of international concern [23]. After its initial discovery, SARS-CoV-2 rapidly spread all over the world, with over 6.2 million confirmed cases COVID-19 and more than 370,000 deaths as of June 1, 2020 [24]. Moreover, recent researches showed that cancer patients were at increased risk of developing COVID-19 with worse outcomes than individuals without cancer, and a significant increase of mortality rate in lung cancer patients with SARS-CoV-2 infection was detected [25, 26]. Despite the clinical features of lung cancer patients, with a higher risk to respiratory infections, immunosuppressed patients, and most of them with a previous of COPD and metastatic disease, the underlying mechanisms in the development and poor prognosis of LUAD patients with COVID-19 are still unclear.

In this study, JAK1 expression in LUAD and the correlations between JAK1 expression and the clinical features and prognosis of LUAD were analyzed using the Oncomine, UALCAN, and Kaplan-Meier plotter databases. The potential molecular mechanism of JAK1 was determined by conducting a GSEA using the Cancer Genome Atlas Lung Adenocarcinoma (TCGA-LUAD) dataset. The association between JAK1 and tumor immune cell infiltration was investigated using the TIMER and CIBERSORT databases. In addition, the JAK1 expression and proportions of immune cells in patients with LUAD with SARS-Cov-2 infection were analyzed. The present study revealed the potential role of JAK1 in LUAD immunology and its prognostic value, which will enhance the understanding on the underlying mechanisms of LUAD.

## RESULTS

### Decreased expression of JAK1 in LUAD

**Figure 1:**
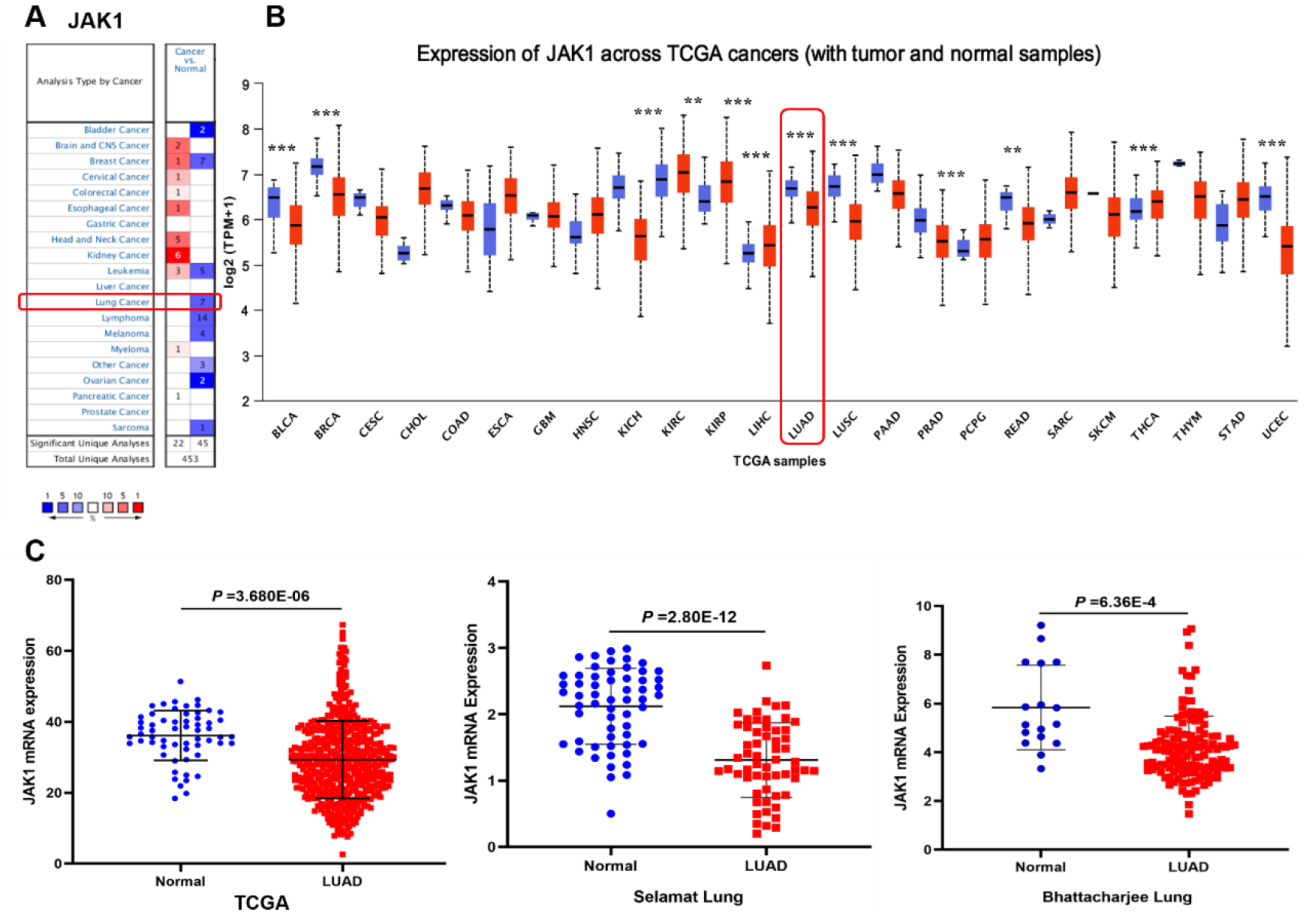
**Expression level of JAK1 in different human cancers.** (**A**) Increased or decreased expression of JAK1 in datasets of different types of cancers compared with normal tissues in the Oncomine database. (**B**) JAK1 expression levels in different cancers from the TCGA database detected by using the UALCAN database. Bule represents Normal, Red represents Tumor. (**C**) Comparison of JAK1 expression levels between LUAD tissues and corresponding normal tissues in different datasets including TCGA, Selamat lung, and Bhattacharjee lung datasets.

The expression levels of JAK1 in tumor and adjacent normal tissues of various cancers were analyzed based on the Oncomine database. As shown in Figure 1A, the result revealed that the expression levels of JAK1 were lower in lung cancer tissues than in normal lung tissues. In addition, the expression levels of JAK1 mRNA were higher in brain and CNS, cervical, colorectal, esophageal, head and neck, kidney, and myeloma cancers. To further assess the expression of JAK1 in multiple malignancies, we analyzed the expression levels of JAK1 mRNA in various types of cancer using the UALCAN database. The expression level of JAK1 was higher in clear cell renal cell carcinoma, papillary renal cell carcinoma, and thyroid carcinoma. By contrast, the expression of JAK1 was significantly decreased in bladder carcinoma, breast carcinoma, colon adenocarcinoma, chromophobe renal cell carcinoma, liver hepatocellular carcinoma, LUAD, lung squamous cell carcinoma, prostate cancer, and adenocarcinoma of the rectum (Figure 1B). These data show that JAK1 has a differential expression and may be regarded as an oncogenic gene associated with the development of various tumors. As shown in Figure 1C, the quantitative evaluation of the expression level of JAK1 in LUAD samples from RNA-seq data of TCGA and microarray data showed that the JAK1 mRNA expression level in LUAD tissues was significantly lower than that in normal tissues. Overall, these results suggest that the expression of JAK1 is decreased in various types of cancer, particularly in LUAD, demonstrating that JAK1 may suppress LUAD tumorigenesis.

### Associations between JAK1 and clinical characteristics of LUAD patients

**Figure 2:**
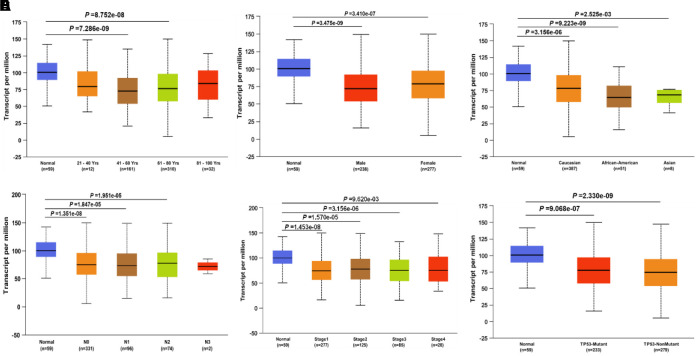
**JAK1 transcription in subgroups of patients with LUAD, divided based on gender, age, ethnicity and other clinical features.** (**A**) age. (**B**) gender. (**C**) ethnicity. (**D**) Lymph -node status. (**E**) Disease stage. (**F**) TP53 mutation status. Color images are available online.

Since the JAK1 expression was significantly decreased in LUAD samples compared with that in the adjacent normal tissues, we focused on the detection of JAK1 expression in LUAD patients with different conditions by conducting further UALCAN database analysis. Results showed that JAK1 expression was much lower in patients with LUAD (aged 41–60 years and 61–80 years) than in normal individuals (Figure 2A). After conducting further analysis of the expression of JAK1 based on gender and race, we found that JAK1 was highly associated with sex and race, and the LUAD male patients had the lowest JAK2 expression levels (Figure 2B, 2C). Another significant clinical feature was the influence of nodal metastasis and individual cancer stages on JAK1 expression. Results indicated that JAK1 expression was closely associated with cancer stage and nodal metastasis (Figure 2D, 2E). A differential analysis of the expression of JAK1 based on TP53 mutation status was also conducted. We found that patients with LUAD with a TP53 mutation status had lower JAK1 expression (Figure 2F). All of these results indicated that a low JAK1 expression was widely associated with the development of LUAD. The detailed clinical characteristics of patients with LUAD are presented in Table 1.

**Table 1:** Clinical characteristics of patients with lung adenocarcinoma.

**Variable**	**Case, n**	**Percentage (%)**
Age (years)		
21-40	12	2.33
41-60	161	31.26
61-80	310	60.19
81-100	32	6.22
Gender		
Female	277	53.79
male	238	46.21
Ethnicity		
Caucasian	387	86.77
African-American	51	11.44
Asian	8	1.79
Lymph-node status		
N0	331	65.8
N1	96	19.09
N2	74	14.71
N3	2	0.4
Disease stage		
Stage 1	277	53.79
Stage 2	125	24.27
Stage 3	85	16.5
Stage 4	28	5.44
TP53 mutation status		
TP53-mutant	233	45.5
TP53-nonmutant	279	54.5

### Protein expression and DNA methylation levels of JAK1 in patients with LUAD

**Figure 3:**
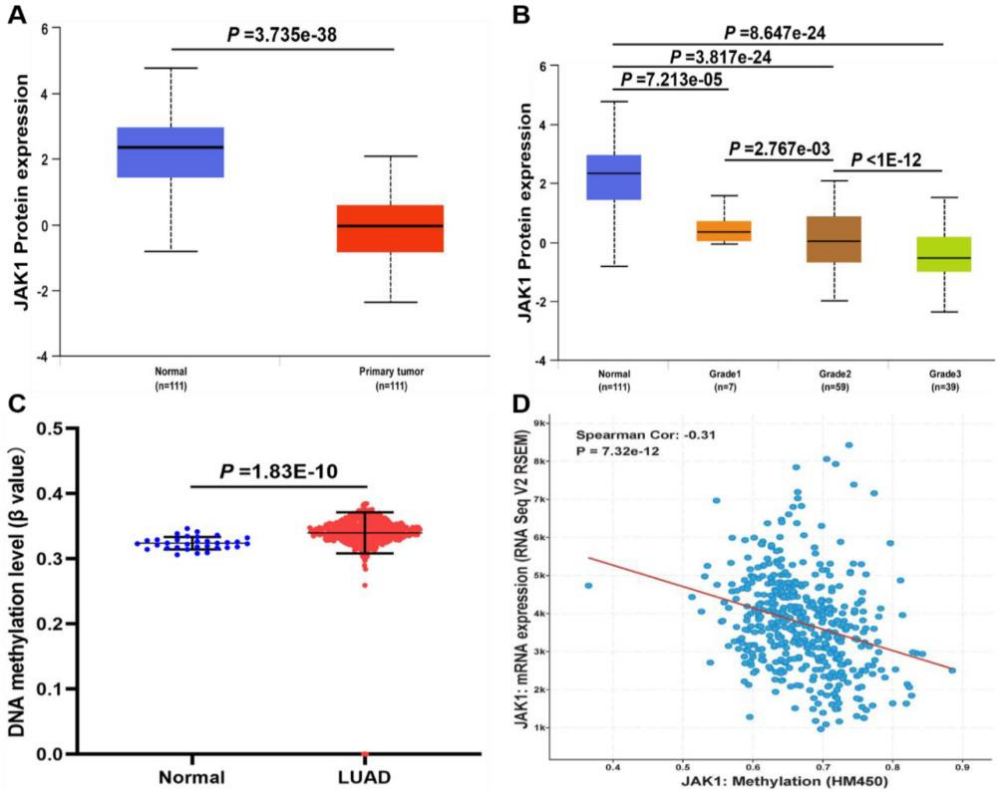
**The protein expression and DNA methylation of JAK1 in patients with LUAD.** (**A**) Protein expression levels of JAK1 in normal and LUAD samples. (**B**) protein expression levels of JAK1 in normal individuals or LUAD patients with tumor grade 1, 2, 3. (**C**) DNA methylation levels of JAK1 in normal individuals and LUAD patients. (**D**) correlation analysis of JAK1 mRNA expression with JAK1 methylation.

To determine whether the JAK1 protein was also differentially expressed in LUAD tissues, a protein expression analysis was conducted using the data from a clinical proteomic tumor analysis consortium in the UALCAN database. Consistent with the RNA expression data, results indicated that the protein expression of JAK1 was lower in LUAD tissues than in normal tissues, and was negatively associated with tumor grade (Figure 3A, 3B). As the tumor grade increased, the expression of JAK1 decreased significantly. These evidences strongly indicated that both mRNA and protein expressions of JAK1 were significantly decreased in LUAD. To further identify the mechanisms underlying the downregulation of JAK1 in LUAD, the methylation levels of JAK1 in the LUAD dataset were analyzed. As shown in Figure 3C, the DNA methylation levels of JAK1 between normal lung samples and LUAD tissues were compared, and a significant difference was observed (p < 0.001). In addition, the gene expression of JAK1 was negatively correlated with its methylation level (Figure 3D). These results revealed that the hypermethylation of JAK1 in patients with LUAD may contribute to downregulation of JAK1 in LUAD; however, this finding requires further investigation in the future.

### Prognostic value of JAK1 in patients with LUAD

**Figure 4:**
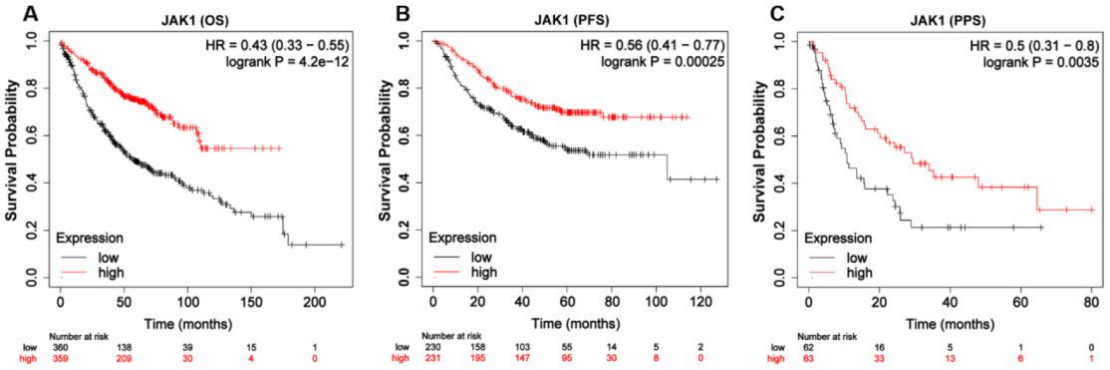
**Kaplan-Meier survival curves of LUAD patients according to the mRNA expression of JAK1 using Kaplan-Meier plotter tool.** (**A**) Over survival curve of patients with LUAD base on JAK1 expression levels. (**B**) Progression-free survival curve of patients with LUAD base on JAK1 expression levels. (**C**) Post-progression survival curve of patients with LUAD base on JAK1 expression levels.

To explore the prognostic value of JAK1 expression levels in patients with LUAD, the Kaplan–Meier plotter database was used. As shown in Figure 4A, low expression of JAK1 was associated with worse OS in patients with LUAD (OS, hazard ratio [HR] = 0.43, 95% confidence interval [CI] = 0.33–0.55, log-rank p = 4.2e–12). Similarly, low JAK1 expression was significantly associated with reduced progression-free survival (PFS) (Figure 4B, PFS, HR = 0.56, 95% CI = 0.41–0.77, log-rank p = 0.00025). Furthermore, low expression of JAK1 mRNA also indicated adverse PPS (Figure 4C). Overall, these results suggest that JAK1 expression is an important factor affecting the survival of patients with LUAD.

### Identification of JAK1-associated signaling pathways using gene set enrichment analysis

**Figure 5:**
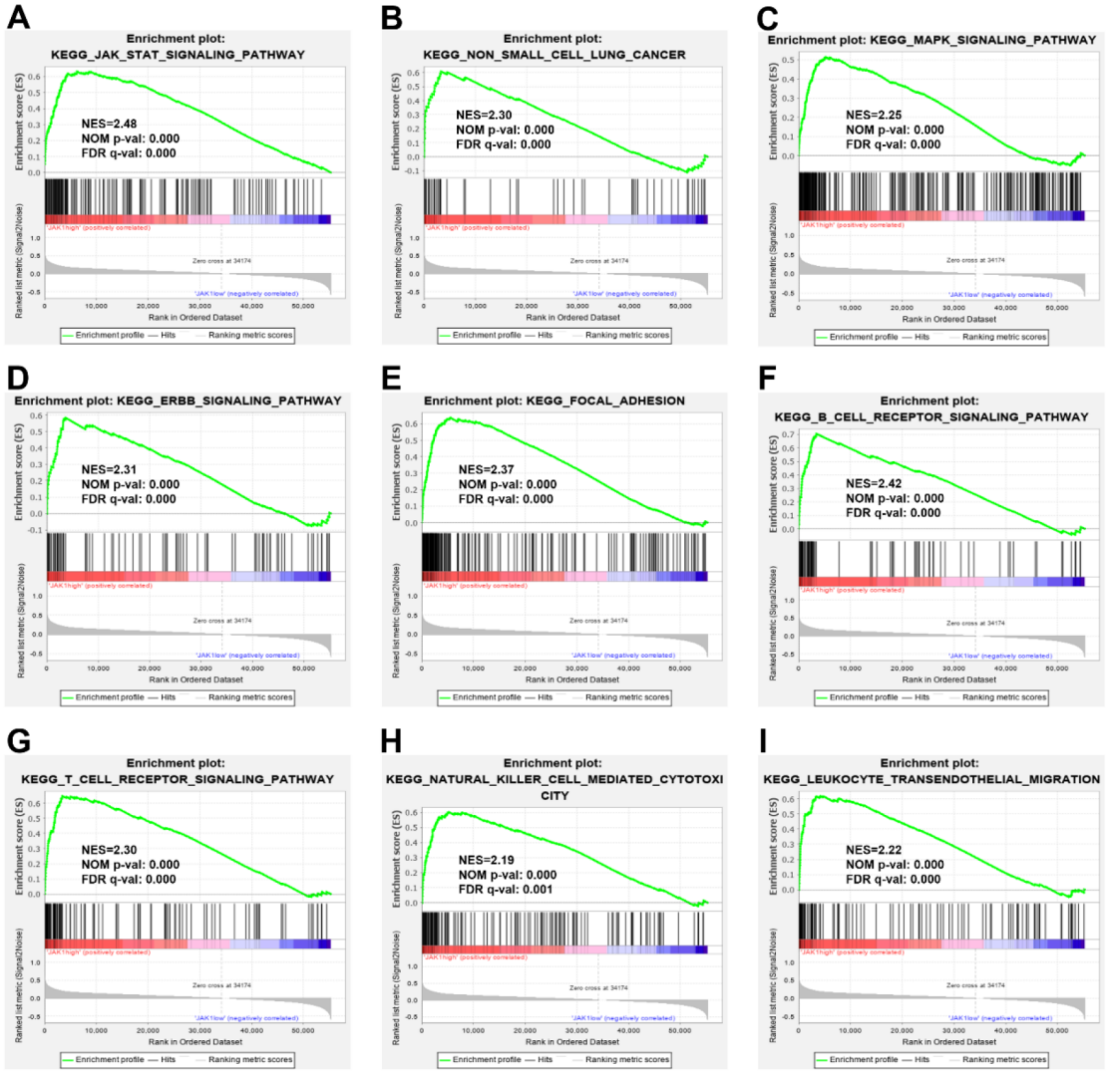
**Enrichment plots from GSEA.** GSEA results indicated that the JAK-STAT signaling pathway (**A**), the non-small cell lung cancer (**B**), the MARK signaling pathway (**C**), the ERBB signaling pathway (**D**), the focal adhesion, (**E**), the B cell receptor signaling pathway (**F**), the T cell receptor signaling pathway (**G**), the natural killer mediated cytotoxicity (**H**), and leukocyte transendothelial migration (**I**) were differentially enriched in LUAD samples with high JAK1 expression. NES, normalized enrichment score; NOM p-val, normalized P-value; FDR q-val, false discovery rate q-value; GSEA, Gene Set Enrichment Analysis.

To explore the potential molecular function of JAK1 in LUAD carcinogenesis, GSEA of high- and low-JAK1 expression datasets acquired from the TCGA database was performed to explore the signaling pathways associated with JAK1. Based on the selected cutoff criteria, a total of 64 signaling pathways were significantly enriched; among them, the top 20 signaling pathways were selected and are presented in Table 2. The significantly upregulated pathways enriched in the high JAK1 group involved in cell adhesion and tumorigenesis were as follows: regulation of the actin cytoskeleton, focal adhesion, ERBB signaling pathway, MAPK signaling pathway, Toll-like receptor signaling pathway, and non-small cell lung cancer. Meanwhile, the pathways associated with immune responses were as follows: JAK-STAT signaling pathway, B-cell receptor signaling pathway, T-cell receptor signaling pathway, chemokine signaling pathway, natural killer cell mediated cytotoxicity, and leukocyte transendothelial migration. The enrichment results are presented in Figure 5.

**Table 2:** Gene sets enriched in the high JAK1 expression phenotype group.

**Gene set name**	**Size**	**NES**	**NOM p-val**	**FDR q-val**
KEGG_JAK_STAT_SIGNALING_PATHWAY	155	2.48	0	0
KEGG_B_CELL_RECEPTOR_SIGNALING_PATHWAY	75	2.42	0	0
KEGG_FOCAL_ADHESION	199	2.37	0	0
KEGG_ERBB_SIGNALING_PATHWAY	87	2.31	0	0
KEGG_T_CELL_RECEPTOR_SIGNALING_PATHWAY	108	2.3	0	0
KEGG_NON_SMALL_CELL_LUNG_CANCER	54	2.3	0	0
KEGG_REGULATION_OF_ACTIN_CYTOSKELETON	213	2.28	0	0
KEGG_PHOSPHATIDYLINOSITOL_SIGNALING_SYSTEM	76	2.28	0	0
KEGG_GLIOMA	65	2.26	0	0
KEGG_NOD_LIKE_RECEPTOR_SIGNALING_PATHWAY	62	2.26	0	0
KEGG_FC_EPSILON_RI_SIGNALING_PATHWAY	79	2.25	0	0
KEGG_MAPK_SIGNALING_PATHWAY	267	2.25	0	0
KEGG_CHEMOKINE_SIGNALING_PATHWAY	188	2.25	0	0
KEGG_COLORECTAL_CANCER	62	2.23	0	0
KEGG_LEUKOCYTE_TRANSENDOTHELIAL_MIGRATION	116	2.22	0	0
KEGG_CHRONIC_MYELOID_LEUKEMIA	73	2.21	0.002	0.001
KEGG_TOLL_LIKE_RECEPTOR_SIGNALING_PATHWAY	102	2.21	0	0.001
KEGG_ACUTE_MYELOID_LEUKEMIA	57	2.2	0.002	0
KEGG_NATURAL_KILLER_CELL_MEDIATED_CYTOTOXICITY	132	2.19	0	0.001
KEGG_SMALL_CELL_LUNG_CANCER	84	2.19	0	0.001

NES: Normalized Enrichment Score; NOM: Nominal; FDR: False Discovery Rate. Gene sets with NOM p-value < 0.05 and FDR q-value < 0.1 are considered as statistically significant.

### Association between JAK1 and tumor-infiltrating immune cells in LUAD

**Figure 6:**
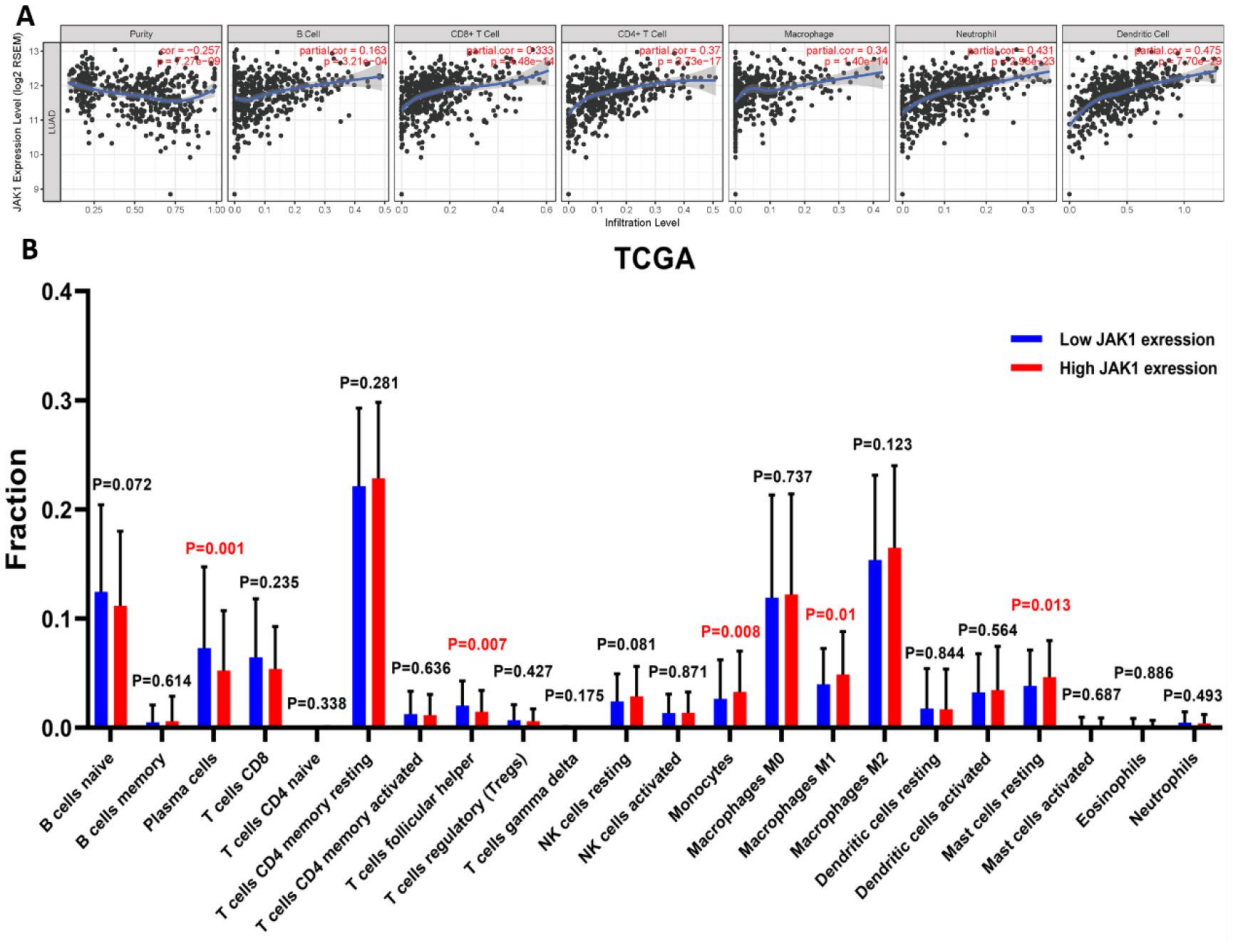
**Correlation analysis of JAK1 expression with immune filtration level in patients with LUAD using TIMER and CIBERSORT databases.** **(A)** JAK1 correlation with tumor purity, B cell, CD8+ T cell, CD4+ T cell, macrophage, neutrophil, dendritic cell in LUAD. (**B**) Proportions of the 22 tumor-infiltrating immune cell types in high and low JAK1 expression groups.

Considering that JAK1 is a crucial mediator of immune cell activation, a correlation analysis between JAK1 expression and six types of immune-infiltrating cells, including B cells, CD8+ T cells, CD4+ T cells, macrophages, neutrophils, and dendritic cells, was performed. As shown in Figure 6A, JAK1 expression levels were significantly positively correlated with infiltrating levels of B cells (r = 0.163, p = 3.21e–04), CD8+ T cells (r = 0.333, p = 4.48e–14), CD4+ T cells (r = 0.37, p = 3.73e–17), macrophage (r = 0.34, p = 1.4e–14), neutrophils (r = 0.431, p = 2.98e–23), and dendritic cells (r = 0.475, p = 7.70e–29) in LUAD. Taken together, the above evidence indicates that JAK1 may be involved in the immune response of patients with LUAD by affecting the immune cells.

Next, we determined whether the immune infiltration status in patients with LUAD was changed between the high and low JAK1 expression groups. The high and low JAK1 expression samples among 497 TCGA LUAD samples were divided into high and low expression groups, respectively. The fraction of the 22 subpopulations of immune cell types in the high and low JAK1 expression groups was clearly determined from the results of the CIBERSORT analysis. Results showed that significantly decreased monocyte and macrophage M1 were observed in the low JAK1 expression group from the TCGA database (Figure 6B).

Moreover, JAK1 was positively correlated with the infiltrating level of macrophages (Figure 6A). The CIBERSORT analysis further revealed that M1 macrophages were significantly upregulated in the high JAK1 expression group, whereas M2 macrophages showed no significant change (Figure 6B). To further analyze the association between JAK1 and different macrophages, the correlations between JAK1 and the markers of M1 macrophages in LUAD were investigated using the TIMER database. Results showed that the M1 macrophage markers such as PTGS2, IRF5, and NOS2 were significantly associated with the expression levels of JAK1 (Supplementary Figure 1). These results indicate that JAK1 may have a potential role in the regulation of the polarization of M1 macrophages. Based on these results, we analyzed the prognostic value of JAK1 expression in LUAD based on the macrophages using the Kaplan–Meier database. As indicated in Supplementary Figure 2A, 2B, low JAK1 expression was significantly associated with unfavorable survival prognosis of LUAD in enriched macrophages (HR = 0.55), whereas a significant difference was not observed in the reduction of the number of macrophages (p > 0.05). Altogether, these results indicate that JAK1 may be involved in the immune response, leading to a worse prognosis in patients with LUAD.

### Determining whether SARS-CoV-2 infection may reduce the expression of JAK1

**Figure 7:**
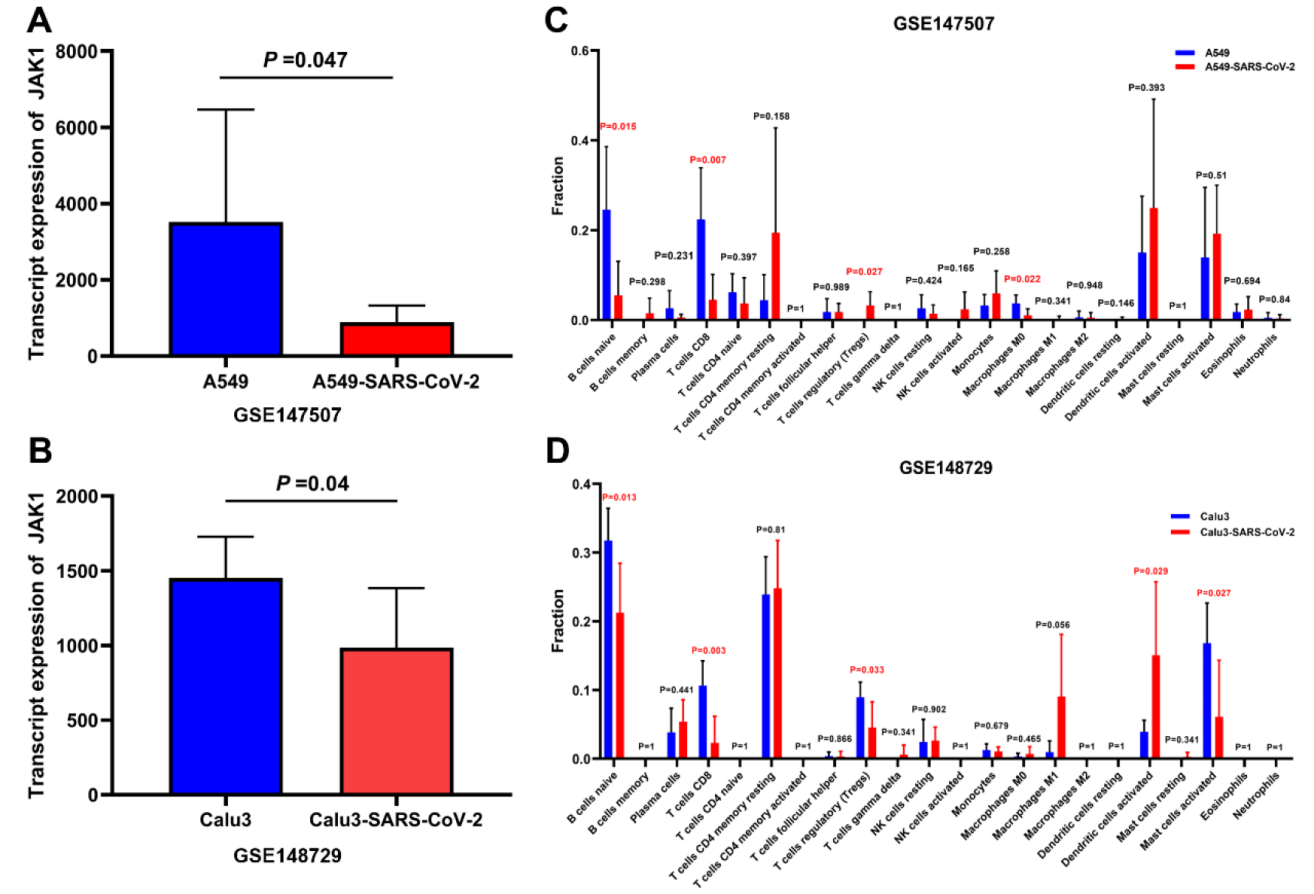
**Analysis of the JAK1 expression and immune cells proportion in LUAD cell lines with SARS-CoV-2 infection.** (**A**) JAK1 expression in A549 cells and A549 cells with SARS-CoV-2 infection in GSE147507. (**B**) JAK1 expression in Calu3 cells and Calu3 cells with SARS-CoV-2 infection. (**C**) Proportions of the 22 tumor-infiltrating immune cell types in A549 cells with and without SARS-CoV-2 infection. (**D**) Proportions of the 22 tumor-infiltrating immune cell types in Calu3 cells with and without SARS-CoV-2 infection.

To further explore the changes in the expression of JAK1 in LUAD cells after SARS-CoV-2 infection, the GSE147507 and GSE148729 cell lines were selected to analyze the changes in JAK1 expression in different cell lines of LUAD after SARS-CoV-2 infection. As shown in Figure 7A, 7B, the expression of JAK1 in SARS-CoV-2 infected A549 cells and Calu3 cells was significantly decreased compared with that in the control group. This finding revealed that JAK1 expression may decrease in LUAD cell lines after SARS-CoV-2 infection. Next, we explored whether the infiltration of multiple immune cells in LUAD was related to SARS-CoV-2 infection. The A549 and Calu3 samples from the GSE147507 and GSE148729 groups were divided into normal and SARS-CoV-2 infection groups. We found a significant decrease in the proportion of CD8+ T cells in the SARS-CoV-2 infection group from the potted database in the GSE147507 (Figure 7C) and GSE148729 samples (Figure 7D).

## DISCUSSION

LUAD is one of the common subtypes of lung malignant neoplasms. Although the diagnostic and therapeutic strategies for LUAD have greatly improved over the past decades, the prognosis of patients with LUAD remains poor. The abnormal gene expression is involved in tumorigenesis and correlated with the prognosis of these patients [27, 28]. However, the potential role of JAK1 in tumor immune infiltration and the prognosis of LUAD remains ambiguous. In the present study, we demonstrated that JAK1 expression was remarkably decreased in LUAD tissues and was associated with gender, ethnicity, age, sex, lymph node status, disease stage, and TP53 mutation status in LUAD patients. DNA methylation is an important form of epigenetic modification of DNA, which can change the genetic expression without changing the DNA sequence, especially in hypermethylation status [29]. The methylation level of JAK1 was significantly increased and was negatively associated with JAK1 gene expression, indicating that the hypermethylation of JAK1 may cause a significant decrease in JAK1 expression. Moreover, low JAK1 expression was significantly associated with unfavorable prognosis. These results suggest that JAK1 may be involved in lung carcinogenesis and affects its prognosis.

Previous studies have reported that JAK1 is highly associated with tumorigenesis and plays important roles in the proliferation, differentiation, and apoptosis of cancer cells [13, 30]. However, the effect of JAK1 on cell proliferation and apoptosis remains unclear. JAK1 was associated with the poor survival of patients with breast cancer; JAK1 knockdown by siRNA or inhibitors can lead to the arrest of cell grow and apoptosis [20, 31]. The abnormal expression of JAK1 was observed in multiple tumor tissues. This finding indicated that JAK1 may be involved in tumorigenesis. JAK1 was significantly decreased in patients with LUAD, which means that JAK1 may play a role in the development of LUAD.

To further investigate the molecular functions and potential mechanisms of JAK1 in LUAD, the GSEA was conducted to explore the pathways enriched in high JAK1 expression samples and identify the top 20 upregulated pathways associated with cell adhesion, tumorigenesis, and the immune response. The significantly enriched pathway related to cell adhesion included the regulation of the actin cytoskeleton and focal adhesion. Perturbation of the actin cytoskeleton is involved in the impairment of the mitochondria and apoptosis, which results in the inhibition of liver cancer cell growth [32]. The upregulated molecules linked to the migratory signals of the actin cytoskeleton promote the invasion and metastasis of cancer cells [33]. Tumorigenesis-associated pathways, such as the ERBB signaling pathway, MAPK signaling pathway, Toll-like receptor signaling pathway, and non-small cell lung cancer, were also predicted in the high JAK1 expression group. In addition, the signaling pathways enriched in the high JAK1 group involved in immune responses were JAK-STAT signaling pathway, B-cell receptor signaling pathway, T-cell receptor signaling pathway, chemokine signaling pathway, natural killer cell-mediated cytotoxicity, and leukocyte transendothelial migration. Chen et al. found that JAK1 is positively correlated with immune cell infiltration, in which the dysregulation of JAK1 was associated with infiltrating macrophages in breast cancer [20]. Overall, these results suggest that JAK1 is highly associated with tumorigenesis and immune-related pathways. Therefore, JAK1 may play a role in the carcinogenesis of LUAD by affecting these signaling pathways, thus resulting in a poor prognosis in patients with LUAD.

The results of TIMER analyses showed that JAK1 was positively associated with the infiltrating levels of CD4+ T cells, CD8+ T cells, macrophages, and dendritic cells. The estimated fraction of 22 tumor-infiltrating immune cells in LUAD was analyzed using the CIBERSORT database. The results indicated that the proportions of monotypes and M1 macrophages were significantly decreased with reduction of JAK1 expression. These results suggest that JAK1 expression is closely associated with the expression of tumor-infiltrating immune cells and may be involved in the immune response to LUAD development, resulting in a poor prognosis in patients with LUAD.

Notably, the results of CIBERSORT analysis showed that the proportion of M1 macrophages in the low JAK1 expression group was significantly decreased compared with that in the high JAK1 expression group. By contrast, no significant differences were observed in the expression of M2 macrophages. To further investigate the association between JAK1 expression and M1 macrophages, the correlation between JAK1 and the marker genes of M1 macrophages was analyzed. Results showed that JAK1 has a significantly positive association with the gene markers of M1 macrophages. This evidence suggests that low JAK1 expression may downregulate the polarization of macrophages to M1 macrophages, which could contribute to the development of LUAD. M1 macrophages produce reactive oxygen and promote a Th1 response with strong tumoricidal activity [34]. A previous study reported that promoting the polarization of M1 macrophages could inhibit the proliferation, invasion, migration, and angiogenesis of tumor cells; facilitate apoptosis; and M1macrophage densities in the tumor islets, stroma, orislets and stroma were positively associated with NSCLC patient’s survival time [35, 36]. It is possible that low JAK1 expression reduces the polarization of M1 macrophages, thus accelerating the progression of LUAD and leading to a poor prognosis.

The outbreak of COVID-19 worldwide has led to a large number of human deaths and severe economic recession [24]. Data from China thus far have shown that mechanical ventilation or ICU admission is more frequently required in cancer patients with COVID-19 infection than in the general population, and higher mortality rate and severity was observed in lung cancer patients with COVID-19 [36]. Decreased JAK1 expression in LUAD patients was associated with unfavorable prognosis, but it was not clear that whether JAK1 was associated with increased mortality rate and severity in LUAD patients with COVID-19. To explore the potential role of JAK1 in the development of LUAD after SARS-CoV-2 infection, the GSE147507 and GSE148729 were used. Results showed that the expression levels of JAK1 decreased in both cell lines after SARS-CoV-2 infection. Elevated serum of interferon (IFN)-γ has been found in patients ARDS in COVID-19, and it exerts multiple effects through the JAK1/JAK2 signaling resulting to the activation of a inflammatory factors downstream cascade [37, 38]. It means that SARS-CoV-2 infection may enhance the production of cytokines and aggravate the pulmonary inflammation through the JAK1 related signaling pathways, thus leading to an increased mortality rate in LUAD patients with COVID-19. We also found that the proportion of CD8+ T cells was also significantly decreased after SARS-CoV-2 infection (Figure 7). JAK1 was involved in the activation of T cells, and its low expression was correlated with a decrease in CD8+ T cells in patients with COVID-19 pneumonia, especially in those with severe and critical cases [39, 40]. These results indicate that SARS-CoV-2 infection may reduce the proportion of CD8+ T cells in LUAD cell lines by inhibiting the expression of JAK1. Lung cancer patients are immunocompromised, especially those receiving antitumor therapy. Some studies have shown that patients with COVID-19 pneumonia have immunodeficiency and hypo-immunity, which may increase the severity of the disease and result in death [41]. Therefore, we focused on JAK1 to explore its possible effect on immune cells in LUAD and thus play a role in the development of this condition. This study is limited by the clinical data of LUAD patients with COVID-19 pneumonia, and further study is needed in the future. However, our findings provide evidence and clues for the treatment and prognosis of patients with LUAD with COVID-19 pneumonia.

In conclusion, JAK1 expression decreased significantly in patients with LUAD, which was correlated with the clinical features and predicted a poor prognosis. JAK1 expression was closely associated with the infiltrating level of immune cells; its low expression reduces the polarization of M1 macrophages. Our study revealed the potential role of JAK1 in tumor immunology and its prognostic value in LUAD. JAK1 might be used as a prognostic biomarker and therapeutic target for LUAD.

## MATERIALS AND METHODS

### Differential expression analysis of JAK1 by oncomine and UALCAN database

Oncomine database (http://www.oncomine.org) is a web-based data open-mining platform for collecting, standardizing, analyzing the microarray data of different human cancers [42]. Besides, the UALCAN database (http://ualcan.path.uab.edu/analysis/) includes the mRNA sequencing data for 32 cancer types from TCGA [43]. In this study, we used the Oncomine and UALCAN database to study the difference of JAK1 expression between different tumors and adjacent normal samples. The threshold of Oncomine was set as follows: p-value =0.001, fold change =1.5, gene ranking =all, and the statistical significance of JAK1 expression was analyzed by Wilcoxon test. To analyze JAK1 expression in LUAD patients with different clinical features, the further UALCAN database analysis was conducted. Furthermore, the JAK1 protein expression data in LUAD were obtained from UALACN databases.

### Differential expression of JAK1 methylation level by TCGA database

To explore the potential mechanism for the down-regulation of JAK1 expression, the methylation data of JAK1 in normal controls and LUAD patients were also obtained from the TCGA database, and the significance was determined by Student’s test. Additionally, the correlation between JAK1 gene expression and its methylation levels data was analyzed by the Spearman method. p-values <0.05 were considered statistically significant.

### Survival analysis of JAK1 expression in LUAD based on Kaplan-Meier plotter database

The Kaplan-Meier plotter database (https://kmplot.com/analysis), contains survival information of 865 patients with LUAD. The prognostic value of JAK1 expression was assessed by overall survival (OS), progression-free survival (PFS), and post-progression survival (PPS) using the hazard ratio (HR), 95% confidence intervals (CI) and log-rank p-value [44]. All patient samples were divided into high expression groups and low expression groups based on the median expression levels of the JAK1. Besides, univariate Cox regression analysis was used to assess the prognostic value of JAK1. In this study, we used the JetSet scores to assess the representation of probe sets, and only the probe sets with the best JetSet scores for JAK1 were selected. The threshold of Cox p and log-rank p was set as 0.05.

### Gene set enrichment analysis (GSEA) of JAK1 in LUAD

To further investigate the potential biological function of the JAK1 in LUAD, GSEA 4.0 was used to evaluate the associations between the high expression and low expression JAK1 groups and tumor-associated pathway in the TCGA lung adenocarcinoma dataset. The reference gene set C2 (c2.cp.kegg.v7.0.symbols.gmt) was selected and the JAK1 expression level was used as a phenotype label. The normalized enrichment score (NES) was acquired by analyzing with 1000 times permutations. The normal p-value <0.05 and the false discovery rate (FDR) <0.05 were set as the cut-off criteria.

### Correlation analysis of JAK1 with immune infiltrating cells in TIMER and CIBERSORT databases

The TIMER database, which contains the infiltration levels of six types of immune cells (B cells, CD4+ T cells, CD8+ T cells, neutrophils, macrophages, and dendritic cells) in various cancers, is an effective tool to analyze the abundance of tumor-infiltrating immune cell from the target gene expression data [45]. The associations between JAK1 expression levels and the above-mentioned immune cell infiltration were assessed by the TIMER database. CIBERSORT is a free trial database for evaluated immune cells composition of tissues based on their gene expression profiles [46]. After uploading the expression profiles from the TCGA-LUAD dataset with the standard gene annotations to the CIBERSORT portal, the deconvolution algorithm applying the LM22 signature and permutation =100 was used to calculated the immune cell infiltration. According to the median JAK1 expression values, the available data were split up into low and high JAK1 expression groups to evaluate the differences in the proportion of immune cells. The correlation of JAK1 expression and gene markers of M1 macrophages in LUAD was analyzed in the TIMER database. P-value <0.05 was set as the screening criterion.

### The JAK1 expression and proportions of immune cells in LUAD cell lines with SARS-CoV-2 infection

GEO (https://www.ncbi.nlm.nih.gov/geo/) is an open-access database that contains various microarrays and high-throughput functional genomics data. To further analyzed the expression levels of JAK1 in LUAD after SARS-CoV-2 infection, the expression profile of GSE147507 and GSE148729 was finally obtained in the GEO database. The GSE147507 database which contained 6 SARS-CoV-2 infected A549 cell samples and 6 control A549 cell samples were based on the GPL18573 platform (Illumina NextSeq 500). Besides, the GSE148729 database which included 6 SARS-CoV-2 infected Calu3 cell samples and 6 control Calu3 cell samples were based on the GPL18573 platform (Illumina NextSeq 500). The selection criteria of samples in GSE147507 and GSE148729 were set as follows: LUAD cell lines [sample type], SARS-CoV-2 [virus infection], RNA-seq data [data type], and p-value was set as 0.05.

### Statistical analysis

A two-tailed Student’s test was used to analyze the expression data in different clinicopathological groups and SARS-CoV-2 infected groups. Overall survival (OS), progression-free survival (PFS), and post-progression survival (PPS) were determined using Kaplan-Meier analysis and a log-rank test. Spearman’s correlation analysis was used to evaluate the linear relationship between the JAK1 mRNA expression levels and the immune cell infiltration level. All statistical tests and charts in this study were conducted using SPSS software (version 22.0; IBM Corp.). p-values <0.05 were considered statistically significant. All graphics were integrated and performed using GraphPad Prism 8.

## Supplementary Material

Supplementary Figures
