# Supplementary material for: Decreased expression of JAK1 associated with immune infiltration and poor prognosis in lung adenocarcinoma
Source: Aging (Albany NY). 2020 Dec 15;13(2):2073–88. doi: 10.18632/aging.202205 (PMC7880401; doi:10.18632/aging.202205)
Supplement: Supplementary Figures [file aging-13-202205-s001.pdf]

SUPPLEMENTARY FIGURES

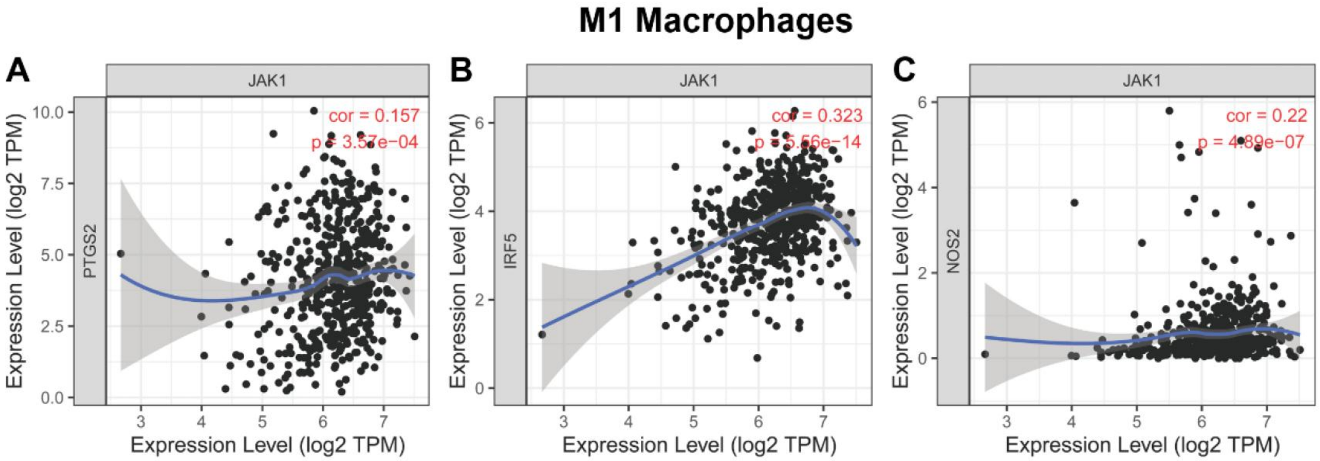

Supplementary Figure 1. Correlation of JAK1 expression with M1 macrophages polarization in LUAD. Scatterplots of the correlation between JAK1 expression and gene markers of M1 macrophages. (**A**) PTGS2. (**B**) IRF5. (**C**) NOS2.

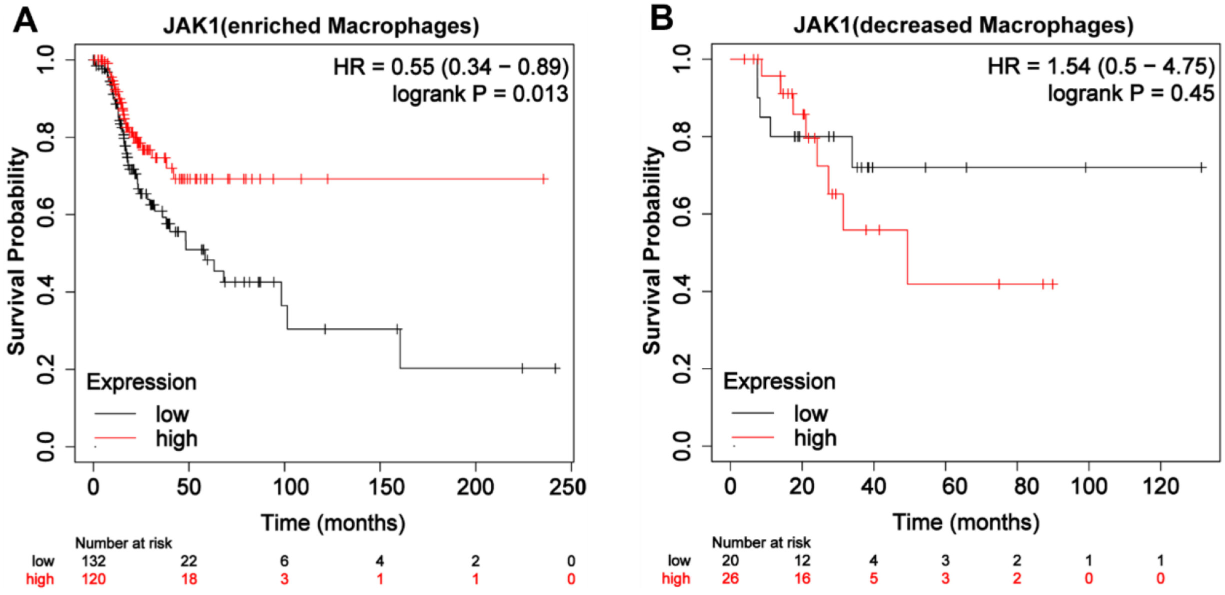

Supplementary Figure 2. Prognostic value analysis of JAK1 expression in LUAD patients based on the macrophages. (**A**) Overall survival of JAK1^{high} and JAK1^{low} LUAD patients with enriched Macrophages analyzed in Kaplan-Meier potter. (**B**) Overall survival of JAK1^{high} and JAK1^{low} LUAD patients with decreased Macrophages analyzed in Kaplan-Meier potter.
